# Climate change and marine life

**DOI:** 10.1098/rsbl.2012.0530

**Published:** 2012-07-11

**Authors:** Anthony J. Richardson, Christopher J. Brown, Keith Brander, John F. Bruno, Lauren Buckley, Michael T. Burrows, Carlos M. Duarte, Benjamin S. Halpern, Ove Hoegh-Guldberg, Johnna Holding, Carrie V. Kappel, Wolfgang Kiessling, Pippa J. Moore, Mary I. O'Connor, John M. Pandolfi, Camille Parmesan, David S. Schoeman, Frank Schwing, William J. Sydeman, Elvira S. Poloczanska

**Affiliations:** 1 Climate Adaptation Flagship, CSIRO Marine and Atmospheric Research, Ecosciences Precinct, GPO Box 2583, Brisbane, Queensland 4102, Australia; 2 Centre for Applications in Natural Resource Mathematics (CARM), School of Mathematics and Physics, The University of Queensland, St. Lucia, Queensland 4072, Australia; 3 Global Change Institute, School of Biological Sciences, The University of Queensland, St. Lucia, Queensland 4072, Australia; 4 Australian Research Council Centre of Excellence for Coral Reef Studies, School of Biological Sciences, The University of Queensland, St. Lucia, Queensland 4072, Australia; 5 National Institute of Aquatic Resources, Technical University of Denmark, Charlottenlund, Denmark; 6 Department of Biology, The University of North Carolina at Chapel Hill, Chapel Hill, NC 27599, USA; 7 Scottish Association for Marine Science, Scottish Marine Institute, Oban, Argyll PA37 1QA, UK; 8 Department of Global Change Research, IMEDEA (UIB-CSIC), Instituto Mediterráneo de Estudios Avanzados, 07190 Esporles, Mallorca, Spain; 9 The UWA Oceans Institute, University of Western Australia, 35 Stirling Highway, Crawley 6009, Western Australia, Australia; 10 National Center for Ecological Analysis and Synthesis, 735 State Street, Suite 300, Santa Barbara, CA 93101, USA; 11 Museum für Naturkunde, Leibniz Institute for Research on Evolution and Biodiversity, Invalidenstrasse 43, 10115 Berlin, Germany; 12 Institute of Biological, Environmental and Rural Sciences, Aberystwyth University, Aberystwyth SY23 3DA, UK; 13 Department of Zoology, University of British Columbia, Vancouver, British Columbia, CanadaV6T 1Z4; 14 Integrative Biology, University of Texas, Patterson Laboratories 141, Austin, TX 78712, USA; 15 Marine Institute, Faculty of Science and Technology, University of Plymouth, Drake Circus, Plymouth PL4 8AA, UK; 16 Faculty of Science, Health, Education and Engineering, University of the Sunshine Coast, Maroochydore DC, Queensland 4558, Australia; 17 Department of Zoology, Nelson Mandela Metropolitan University, Port Elizabeth, South Africa; 18 Environmental Research Division, Southwest Fisheries Science Center, NOAA Fisheries Service, 1352 Lighthouse Avenue, Pacific Grove, CA 93950-2097, USA; 19 Farallon Institute for Advanced Ecosystem Research, 101 H. Street, Suite Q, Petaluma, CA 94952, USA

**Keywords:** climate change, marine science, detection and attribution

## Abstract

A Marine Climate Impacts Workshop was held from 29 April to 3 May 2012 at the US National Center of Ecological Analysis and Synthesis in Santa Barbara. This workshop was the culmination of a series of six meetings over the past three years, which had brought together 25 experts in climate change ecology, analysis of large datasets, palaeontology, marine ecology and physical oceanography. Aims of these workshops were to produce a global synthesis of climate impacts on marine biota, to identify sensitive habitats and taxa, to inform the current Intergovernmental Panel on Climate Change (IPCC) process, and to strengthen research into ecological impacts of climate change.

## 1. Introduction

Marine ecosystems cover 71 per cent of the Earth's surface, yet our knowledge of their response to climate change is a mere drop in the ocean compared with terrestrial systems [1,2]. In the Intergovernmental Panel on Climate Change (IPCC) Fourth Assessment Report in 2007, less than 1 per cent of the synthesis information on impacts of climate change on natural systems came from marine life [1,3]. However, there is increasing evidence suggesting that marine plants and animals could respond as fast or faster than their terrestrial counterparts, from both observations [3–5] and theory [6]. The need for better coverage of marine systems is compelling, given the ecosystem services provided by the world's oceans and the imminent fifth IPCC assessment report. Here, we describe the major advances from the National Center of Ecological Analysis and Synthesis (NCEAS) workshop series, and discuss emerging research directions for the discipline of climate change ecology. Specific questions included: (i) Are marine species and communities responding to climate change as anticipated? (ii) How do rates of responses compare to terrestrial systems? (iii) Which taxonomic groups and biomes are most sensitive? and (iv) How can we improve the design and execution of climate change ecology studies to strengthen the robustness of conclusions?

## 2. Climate impacts: pervasive, coherent and fast

In the first workshop, we discussed whether our greater understanding of terrestrial climate impacts could be used to fill the gap in knowledge of marine systems. Although there are commonalities, we concluded that many ocean responses are unique, because biology is influenced by the contrasting temporal and spatial scales of oceanic and atmospheric processes. For example, ramifications of slow ocean dynamics imply that decreases in ocean pH, which are likely to impact calcifying organisms, from corals in the tropics to pelagic snails in polar ecosystems, will take tens of thousands of years to re-equilibrate to preindustrial conditions. It also became apparent that detection and attribution of climate change impacts in marine systems pose distinct challenges for marine ecologists. These include sampling in a three-dimensional environment, natural variability at decadal or longer time scales (or potentially marine researchers have more awareness of it than their terrestrial counterparts), cooling of large regions (about 15%, 1960–2009) of the ocean [6], and the inadequate temperature estimates in shallow coastal waters (e.g. the intertidal zone) from global climate models.

To address our five major questions, we conducted a comprehensive meta-analysis of the published literature on impacts of climate change on marine life. This required collecting information on the spatial and temporal extent of each study, the statistical approaches applied, whether there were changes in local or global climate metrics, and the robustness of published conclusions. Over the course of the next several workshops, we reviewed the climate change literature and compiled a global database containing 1736 observations from 209 published studies on 857 species. Interpretation of individual studies was discussed in detail within the workshops.

Although there is a perception in the general public that impacts of climate change on ocean ecosystems are an issue for the future, we were stunned by the pervasive nature of changes already observable across various taxa and oceans. We found that climate change was having a coherent and significant impact across all ecosystems (coastal to open ocean), latitudes (polar to tropical) and trophic levels (phytoplankton to top predators). Observed rates of change in phenology and distribution were faster than those observed from terrestrial plants and animals. This generated discussion as to the vulnerability of species and ecosystems. Tropical species may be particularly vulnerable to climate change due to narrow physiological limits in both terrestrial and marine systems. Therefore, hyper-diverse regions, such as the Indo-Pacific marine biodiversity hotspot, may be under threat.

## 3. Data gaps: more commitment for time series

**Figure 1.**
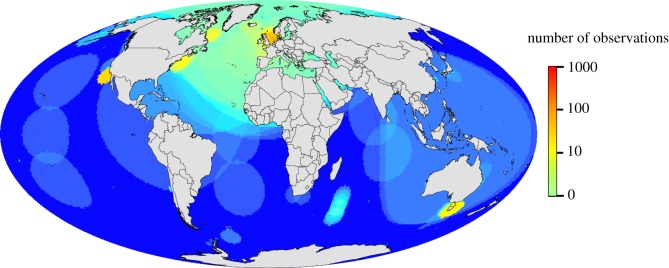
A density plot of marine biological time series more than 19 years in length used to assess climate change impacts.

A major limitation of global analyses is that research effort is extremely patchy in space. Analysis of the assembled database revealed that, as with terrestrial systems, our knowledge of climate impacts was dominated by a few well-studied regions (figure 1). In particular, temperate regions of the North Atlantic and North Pacific are relatively well covered by time series, especially the North Sea. There are few long-term observations in the vast waters of the tropics and subtropics, around developing nations, and those in the Southern Hemisphere, and in particular in the Indian and Southern Oceans. With respect to different habitats in our database, we anticipated capturing most observations from the best studied and understood taxa. By far, the most studied group in terms of time series in the world is fish. This group accounted for 41 per cent of our observations in the database, but there are undoubtedly many more fisheries time series that have not been analysed in a climate change context. Workshop participants were also surprised to find that there were not more observations of coral reefs that passed our database criteria, such as long-term combined observations of biological and climate variables. This is despite the strong attribution link to temperature based on solid mechanistic understanding of the role that warming plays in coral bleaching events, the success of satellite prediction programmes based on temperature and the palaeontological evidence.

The workshops discussed many ways to rectify the lack of long-term observations in sparsely sampled ocean regions. A powerful approach that is increasingly being applied involves measuring changes in coral growth from cores, which extend back centuries and can help fill data gaps in the tropical ocean. We also recognized that the physical oceanographic community had been successful in obtaining ongoing funding for climate change observations. The Global Ocean Observing System, a global initiative supporting marine observations for research and industry, has historically focused on physical properties of the ocean, but is increasingly including biology. There is an emerging opportunity for biologists to work closely with physical oceanographers to form integrated observing systems, thereby enabling a critical mass of researchers to lever funding. A good example is the Australian Integrated Marine Observing System, which, in addition to standard physical measurements, collects regular data on plankton, benthos and fish from multiple platforms and often in conjunction with physical variables. The Ocean Biogeographic Information System could also potentially be used for targeted climate change research. This massive online database currently has 32.3 million occurrence records. The challenge is standardizing these data to make robust time series, but the payoff would be well worth the effort. An important part of the solution to filling the global gaps in time series is the identification of funding mechanisms that can support ocean observations in developing nations.

## 4. Effects of warming: an ecological viewpoint

By the fourth workshop, it had become clear that we needed to develop our general hypotheses for climate change responses beyond earlier spring shifts and poleward range shifts. We reviewed multiple examples where responses to climate change did not follow these general expectations. For example, species' range expansions sometimes followed the east–west axis of coastlines (not poleward), or peaks of timing of breeding of seabirds occurred later (not earlier) in the year. In almost all such cases, studies-related species' responses to changes in a local climate variable. We discussed the challenges that a changing thermal environment pose for species and the need for ecologically relevant measures of temperature change. We applied the concept of the velocity of climate change to sea surface temperature datasets, to produce predictions for the pace and direction that species should move to track regional climate change [6]. We also devised a new index based on shifts in peak timing of monthly temperatures, to refine hypotheses for changing phenology. These indices give a complex mosaic of predicted range shifts and phenology changes that deviate from simple poleward migration and earlier springs or later autumns. Workshop participants considered the relevance of these indices to species responses and the likely differences between land and ocean species. These indices were also used to identify potential conservation concerns, because areas of high marine biodiversity often have greater velocities of climate change and seasonal shifts.

## 5. Establishing climate change ecology as a discipline

The emerging discipline of climate change ecology has important lessons for public policy and faces intense public scrutiny. It is thus critical that climate change ecology has robust, transparent and defensible scientific approaches. The workshops provided an excellent venue to allow self-critical evaluation of the discipline and to make concrete recommendations for increased rigour.

Participants realized that many papers in the discipline, including their own, had crucial shortcomings. For example, it was sobering that 55 per cent of all biological observations purporting to assess impacts of climate change were not subjected to explicit statistical tests of congruence with a climate variable. The use of correlational analyses to relate biological to physical variables should be the minimum requirement in studies of climate change ecology. Further, in the database, 75 per cent of all relationships between biology and physics included only climate variables and did not consider alternative drivers. These studies could over-estimate the effect of climate change, as they do not partition out other pervasive human pressures; neither do they resolve effects of natural climate variability. Many of the strongest analyses were in fisheries science, where there is a tradition of separating natural climate variability from human exploitation; these analyses provide a template for further work. However, probably the biggest weakness was the absence of prior expectations based on observations, experiments or theory, resulting in a prevalence of less robust post-hoc explanations of observations. Many studies did not document, either up front or even in the discussion, the changes in biology that authors were expecting to see under climate change, or the reasoning behind them. Palaeontological data, experiments and ecological theory need to be better harnessed to generate evidence-based prior expectations. The detection and attribution of climate change responses can be rapidly improved through clear formulation of prior expectations, the use of a range of statistical approaches and inclusion of alternative hypotheses.

## 6. Concluding remarks: next steps in climate change ecology

The series of meetings fostered collaboration among a diverse range of scientists and has delivered new understanding. The NCEAS format has been extremely effective, in part because participants came from a range of career stages, from PhD students to fully tenured Professors. This mixture of career stages not only provided young researchers with excellent learning opportunities, but established researchers also benefitted by collaborating with people who had the time and drive to see ideas through to completion. Further, each five-day meeting was sufficiently long to allow real work to be done and to ensure that participants felt comfortable with each other. NCEAS provided a successful and cost-effective model for doing integrative, interdisciplinary science and similar centres are needed to tackle pressing scientific and environmental issues.

Finally, we believe that major advances in our understanding of climate change ecology will require close collaboration between scientists from around the world working on key primary datasets. Current global studies are meta-analyses of published data. They suffer from both publication bias and a focus on only a single aspect of climate change (e.g. phenology) in each study. They also concentrate on animals and plants that are responding [1,2]. For a more integrated and unbiased view of climate impacts, we need to bring together long-term primary observations on multiple taxa across the globe. A key unanswered question in our working group is whether species not responding to climate change in typical ways are more or less vulnerable than species rapidly changing their distribution and phenology.
